# Immune Profile and MRI-Detected Cardiac Fibrosis and Edema in Hypertensive and Non-Hypertensive Patients with COVID-19

**DOI:** 10.3390/jcm13237317

**Published:** 2024-12-02

**Authors:** Renata Moll-Bernardes, Gabriel C. Camargo, Andréa Silvestre-Sousa, Julia Machado Barroso, Juliana R. Ferreira, Mariana B. Tortelly, Adriana L. Pimentel, Ana Cristina B. S. Figueiredo, Eduardo B. Schaustz, José Carlos P. Secco, Sergio C. Fortier, Narendra Vera, Luciana Conde, Mauro Jorge Cabral-Castro, Denilson C. Albuquerque, Paulo H. Rosado-de-Castro, Martha V. T. Pinheiro, Olga F. Souza, Ronir R. Luiz, Emiliano Medei

**Affiliations:** 1 D’Or Institute for Research and Education, Rio de Janeiro 22281-100, Brazil; renata.moll@idor.org (R.M.-B.); gabccamargo@gmail.com (G.C.C.); andreasilvestre0203@gmail.com (A.S.-S.); machadobarroso.julia@gmail.com (J.M.B.); julianacp2@gmail.com (J.R.F.); mariana.tortelly@niteroidor.com.br (M.B.T.); adriana.munford@niteroidor.com.br (A.L.P.); ana.figueiredo@rededor.com.br (A.C.B.S.F.); eduardo.schaustz@idor.org (E.B.S.); jose.secco@idor.org (J.C.P.S.); sergio.fortier@rededor.com.br (S.C.F.); denilsoncalbuquerque@gmail.com (D.C.A.); paulo.rosado@idor.org (P.H.R.-d.-C.); martha.pinheiro@rededor.com.br (M.V.T.P.); olga.souza@rededor.com.br (O.F.S.); ronir@iesc.ufrj.br (R.R.L.); 2 Evandro Chagas National Institute of Infectious Disease, Oswaldo Cruz Foundation, Rio de Janeiro 21040-360, Brazil; 3 Cardiology and Internal Medicine Department, Rede D’Or São Luiz, Rio de Janeiro 22281-100, Brazil; 4 Institute of Biophysics Carlos Chagas Filho, Federal University of Rio de Janeiro (UFRJ), Rio de Janeiro 21941-902, Brazil; vera.narendra@gmail.com (N.V.); conde_luciana@hotmail.com (L.C.); 5 Institute of Microbiology Paulo de Góes, UFRJ, Rio de Janeiro 21941-902, Brazil; maurojorge@micro.ufrj.br; 6 Department of Pathology, Faculty of Medicine, Fluminense Federal University, Niterói, Rio de Janeiro 24033-900, Brazil; 7 Cardiology Department, Rio de Janeiro State University, Rio de Janeiro 20551-030, Brazil; 8 Institute for Studies in Public Health—IESC, UFRJ, Rio de Janeiro 21941-598, Brazil; 9 National Center for Structural Biology and Bioimaging, UFRJ, Rio de Janeiro 21941-902, Brazil

**Keywords:** CD8+, myocardial fibrosis, T2, extracellular volume, hypertension, immune system, COVID-19

## Abstract

Cardiac involvement in 2019 coronavirus disease (COVID-19) survivors has been reported frequently. An exacerbated immune response may be the main mechanism of myocardial injury and late cardiac sequelae in this population. **Background/Objectives:** We investigated the immune profile in hypertensive and non-hypertensive patients with COVID-19 who developed late cardiac fibrosis and edema, as detected by magnetic resonance imaging (MRI). **Methods:** We evaluated associations of cytokine and immune-cell subset levels during hospitalization for COVID-19 with the presence of myocardial interstitial fibrosis [represented by the extracellular volume (ECV)] or edema (represented by the T2), detected by cardiac MRI examination after discharge, in hypertensive and non-hypertensive patients. **Results:** Patients with hypertension had reduced B-cell percentages, increased natural killer cell percentages, and higher interleukin (IL)-4, IL-5, IL-13, IL-17A, and tumor necrosis factor-β levels compared to patients without hypertension. Larger percentages of human leukocyte antigen DR isotope^+^ blood cells, reflecting CD8+ T-cell activation, correlated with increased T2 and ECV in hypertensive patients. The HLA-DR mean fluorescence intensity was associated with ECV in non-hypertensive patients. **Conclusions:** Our findings reveal cytokine and immune-cell dysregulation in both hypertensive and non-hypertensive patients with COVID-19, along with moderate correlations between CD8+ T-cell activation and increased cardiac MRI markers of myocardial interstitial fibrosis and edema. These results contribute to a deeper understanding of immune dysfunction mechanisms involved in myocardial remodeling.

## 1. Introduction

The 2019 coronavirus disease (COVID-19) has posed one of the most critical health problems in the past century. Although severe acute respiratory syndrome coronavirus 2 (SARS-CoV-2) is classically considered a virus that impairs the respiratory tract, clinical evidence demonstrates that it also damages other organs. The late consequences of SARS-CoV-2 infection in different organs are not completely understood. Survivors of severe COVID-19 may show increased sympathetic neural activity, vascular endothelial dysfunction, and reduced exercise capacity [1], among other issues, leading to significant changes in healthcare [2]. Several cases of myocardial injury during SARS-CoV-2 infection have been described, and cardiac involvement has also been detected later, in patients with symptoms of long COVID-19.

Hypertension is among the comorbidities most frequently associated with worse COVID-19 prognosis. At the beginning of the pandemic, Huang et al. [3] showed that a larger proportion of patients with COVID-19 and hypertension required noninvasive mechanical ventilation compared to those without hypertension, and the mortality rate was higher among hypertensive patients with the disease. Other comorbidities, such as diabetes and obesity, have been associated with increased immune dysregulation and, thus, COVID-19 severity, as highlighted in previous work from our group [4].

The dysregulation of the immune response has been consistently reported as one of the most relevant pathophysiological mechanisms of myocardial involvement in patients with COVID-19. Our group and others have documented deviations in the quantities of immune-cell subsets, as well as elevated levels of circulating cytokines, linked to COVID-19 progression and myocardial injury [5,6,7,8,9,10]. In addition, we previously identified troponin as a valuable mortality risk predictor for patients hospitalized with COVID-19 [11]. We also found that lymphopenia, cluster of differentiation (CD)8+ CD38+ mean fluorescence intensity (MFI), and CD8+ human leukocyte antigen DR isotope (HLA-DR)+ MFI were immune biomarkers of myocardial injury in hypertensive patients with COVID-19 [5].

Puntmann et al. [12] detected cardiac involvement by cardiac magnetic resonance imaging (MRI) in a very large percentage of patients who had recently recovered from COVID-19, independent of disease severity and preexisting conditions. An increasing number of reports describe long COVID symptoms and the possibility of late myocardial involvement [13,14,15,16,17,18]. The mechanism underlying this late involvement is probably related to the exacerbation of the immune response, but it has not been studied fully.

Edema and fibrosis have been reported as components of this late cardiac involvement and may be related to the COVID-19-induced immune response with myocardial injury and inflammation related to previous comorbidities, such as hypertension [19,20]. However, the link between immune profiles and cardiac fibrosis/edema in late COVID-19 is poorly understood. A better comprehension of the associations between immune-system abnormalities and late cardiac involvement of COVID-19, independent of cardiac comorbidities (particularly hypertension), is required. Thus, we assessed the associations of immune-cell and cytokine levels during hospitalization for COVID-19 with the presence of myocardial fibrosis and edema, as detected by MRI performed after discharge, in patients with and without hypertension.

## 2. Materials and Methods

### 2.1. Study Design and Participants

This prospective study was conducted with data from consecutive adult patients hospitalized with confirmed COVID-19 who were referred to the cardiology departments of five tertiary hospitals in Rio de Janeiro, Brazil, due to the presence of myocardial injury (defined by increased troponin levels) or coagulation abnormalities (defined by increased D-dimer levels). SARS-CoV-2 infection was confirmed by real-time reverse-transcription polymerase chain reaction of nasopharyngeal and/or oropharyngeal swab samples. Blood samples were collected during hospitalization for the assessment of biomarkers (troponin, D-dimer, and a panel of 27 cytokines and 19 immune-cell subsets). The patients were followed prospectively after hospital discharge and invited to undergo MRI examinations for the evaluation of cardiac structure and function. Therefore, the MRI was performed post-discharge.

Demographic, clinical, and laboratory data (including those on comorbidities, complications, and treatments) were collected from patients’ electronic medical records and entered into electronic case-report forms using the Research Electronic Data Capture platform (Vanderbilt University, Nashville, TN, USA). Patients were allocated to groups according to age (18–59 and ≥60 years) and history of hypertension.

The study was conducted according to the Helsinki criteria for human research. The institutional review boards and ethics committees of the participating institutions approved the study protocol (CAAE#34035120.1.0000.5249). All patients provided written informed consent before enrollment.

### 2.2. Biomarker Quantification

Plasma samples were separated after centrifugation and stored at −20 °C until analysis. Circulating levels of cytokines were measured using the MILLIPLEX MAP human cytokine/chemokine magnetic bead panel (#HCYTMAG-60K-PX29; Merck Millipore, Billerica, MA, USA) according to the manufacturer’s instructions. The C-reactive protein level was measured by latex-enhanced immunoturbidimetric assay. Cytokines not detected in >50% of the patient samples were excluded from further analyses. Cytokine values are presented as pg/mL. It is important to note that here we have shown the biomarkers level only at presentation.

#### Peripheral Blood Mononuclear Cell Isolation

Blood samples collected into ethylenediaminetetraacetic acid (EDTA) tubes (BD Vacutainer^®^ spray-coated K2EDTA tube; Becton, Dickinson and Company, Franklin Lakes, NJ, USA) were centrifuged at 1500× *g* at 21 °C for 15 min. Lymphocytes and monocytes were quantified by photometry using an automated ABX Micros 60 system (Horiba Medical, Montpellier, France). To obtain peripheral blood mononuclear cells (PBMCs), density gradient centrifugation (Ficoll-Paque; GE Healthcare, Piscataway, NJ, USA) was performed, as described previously [7].

### 2.3. Flow Cytometry

The immune-cell populations were quantified, and the results were presented as percentages. PBMCs (1 × 10^6^) were stained with various combinations of fluorophore-conjugated antibodies, as described previously [7]. The following profiles were quantified: total monocytes, total lymphocytes, B lymphocytes, T lymphocytes, natural killer (NK) cells, CD4+ T cells, CD8+ T cells, CD4/CD8 cell ratio, CD8+ CD38+ T cells (percentage and CD38 MFI), CD8+ HLA-DR+ T cells (percentage and HLA-DR MFI), CD8+ natural killer group 2A (NKG2A)+ T cells (percentage and NKG2A MFI), CD8+ HLA-DR+ CD38– T cells, CD8^+^ HLA-DR+ CD38+ T cells, CD8+ HLA-DR– CD38+ T cells, and CD8+ HLA-DR– CD38– T cells. In addition, the approximate membrane expression (MFI) of the antigens HLA-DR and CD38, important markers of T-cell activation, and of NKG2A, an inhibitory T-cell receptor, was evaluated to improve the understanding of lymphocyte properties.

### 2.4. Cardiac MRI Protocol

#### 2.4.1. Image Acquisition

Cardiac MRI studies were performed using a 3.0-Tesla scanner (Magnetom Prisma; Siemens Healthcare, Erlangen, Germany). Cine images were acquired in long- and short-axis planes using a steady-state free-precession sequence with retrospective gating [21]. Images were acquired before and after the intravenous injection of 0.4 mL/kg gadolinium-DOTA contrast (Dotarem; Guerbet, Aulnay-sous-Bois, France). Delayed enhancement images were obtained with an inversion recovery gradient echo sequence. T1 mapping images were acquired in a mid-ventricular short-axis plane using a modified look-locker inversion recovery sequence before and 15 min after contrast infusion [22,23,24]. A blood sample was collected, and the extracellular volume (ECV) was calculated using the hematocrit value to adjust for contrast volume distribution.

#### 2.4.2. Image Analysis

Cine images were analyzed to assess left-ventricular (LV) morphological and functional parameters [25]. Image analysis was conducted in a blinded manner using commercially available multi-platform software (OsiriX MD version 12.5.0; Pixmeo, Geneva, Switzerland). Qualitative and quantitative characteristics of myocardial replacement fibrosis were evaluated through visual assessment of late gadolinium enhancement (LGE) images. Myocardial T2 and pre- and post-contrast T1 values were quantified directly from T2 and T1 mapping images, respectively, utilizing the mean signal intensity value from manually drawn regions of interest (ROI) covering the entire LV myocardium, avoiding areas of subendocardial or transmural LGE. Areas of nonischemic LGE were included in the ROI if present, as previously suggested [26]. The presence of diffuse interstitial myocardial fibrosis was inferred from myocardial ECVs derived from mean LV myocardial T1 values obtained from pre- and post-contrast T1 maps acquired in a single short-axis plane at the mid-ventricular level [27].

### 2.5. Outcomes

The primary outcomes assessed were correlations of cytokine and immune-cell subset levels during hospitalization with the presence of myocardial fibrosis (based on ECVs) and edema (based on T2 imaging data) after discharge in hypertensive and non-hypertensive patients with COVID-19. Secondary outcomes assessed were differences in cytokine and immune-cell subset levels, ECVs, and T2 markers of edema between hypertensive and non-hypertensive patients.

### 2.6. Statistical Analysis

Categorical variables were characterized as proportions, and continuous variables were described as means ± standard deviations and medians with ranges. The chi-squared test was used to compare the frequencies of clinical variables between groups with and without hypertension. Distribution normality testing was performed using the Kolmogorov–Smirnov test. Continuous variables were compared between hypertensive and non-hypertensive groups using the Mann–Whitney test. Spearman correlation coefficients were calculated for all cytokines and immune biomarkers for hypertensive and non-hypertensive patients. The significance level was set at 5%. All analyses were performed using SPSS software (version 24.0; IBM Corporation, Armonk, NY, USA).

## 3. Results

In total, 180 hospitalized patients were enrolled prospectively in this study between November 2020 and September 2022. Forty-six of these patients (21 with and 25 without hypertension) consented to return for cardiac MRI examination. The patients were predominantly male (73.9%), but the sex distribution did not differ significantly between groups. Relative to non-hypertensive patients, hypertensive patients were older (64.7 vs. 49.1 years; *p* = 0.002), and more of them had diabetes (47.6% vs. 8%; *p* = 0.005). The groups did not differ according to previous cardiac or pulmonary disease, obesity, D-dimer level, or presence of myocardial injury during hospitalization (Table 1). Blood samples for biomarker analysis were collected from 46 patients at a median of 3.0 days after hospitalization. The median interval between hospital discharge and cardiac MRI was 71 days.

### 3.1. Immune Signatures

We explored levels of 45 immune markers during hospitalization. Descriptive statistics are provided in Table 2. Among the cell markers, the total B lymphocyte percentage was decreased (*p* = 0.043) and the NK lymphocyte percentage was increased (*p* = 0.008) in patients with hypertension relative to those without hypertension (Table 3). Relative to non-hypertensive patients, hypertensive patients had higher interleukin (IL)-4 (*p* = 0.048), IL-5 (*p* = 0.020), IL-17A (*p* = 0.010), IL-13 (*p* = 0.034), and tumor necrosis factor (TNF)-β (*p* = 0.013) levels (Table 4).

### 3.2. MRI Findings

**Figure 1:**
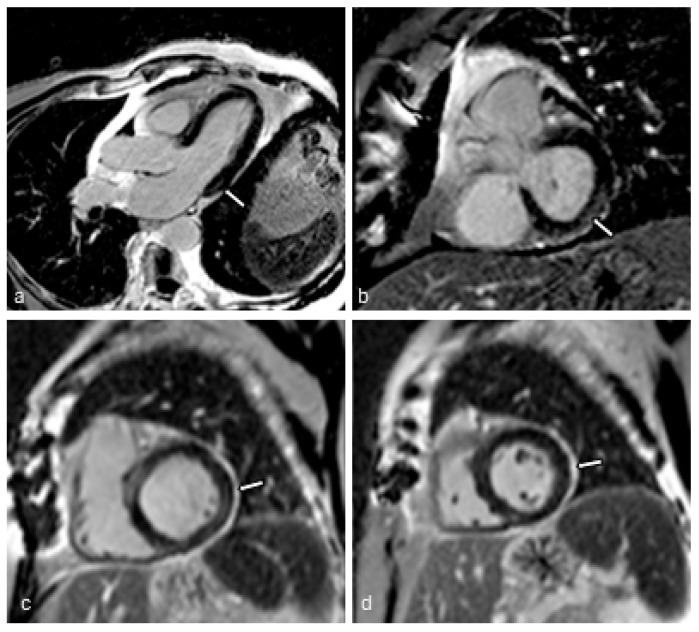
Cardiac MRI images from two COVID-19 survivors. Inversion recovery late gadolinium enhancement (LGE) images in three-chamber (**a**) and short axis (**b**) views of a 71-year-old man without hypertension, depicting mild non-ischemic myocardial LGE at the inferolateral wall, with an end-diastolic volume index (EDVI) of 59 mL/m^2^ and a 62% ejection fraction (EF). (**c**,**d**) Inversion recovery LGE images in short axis views of an 85-year-old woman with hypertension showing pericardial LGE, with an EDVI of 74 mL/m^2^ and a 61% EF.

Eleven (23.9%) patients showed nonischemic LGE, with no difference between hypertensive and non-hypertensive patients (Figure 1a). Five (10.0%) patients, all of whom were hypertensive, showed subendocardial LGE, and three (6.5%) patients, two of whom were hypertensive, had transmural LGE. Sixteen (34.8%) patients had pericardial enhancement, with no difference between groups (Figure 1b).

**Figure 2:**
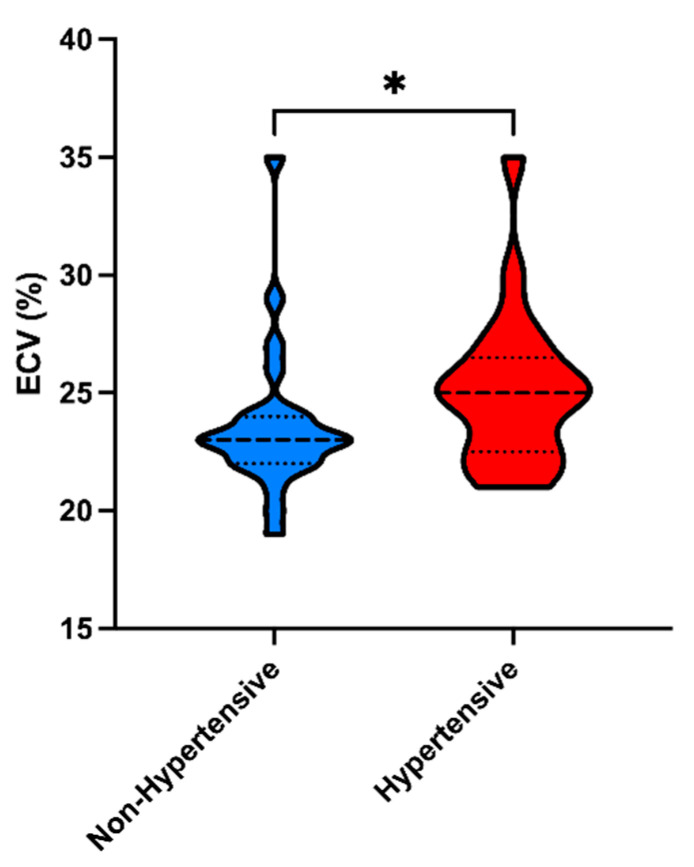
Extracellular volumes (ECVs) in hypertensive and non-hypertensive COVID-19 survivors (median, 25.0 vs. 23.0; * *p* = 0.05).

**Figure 3:**
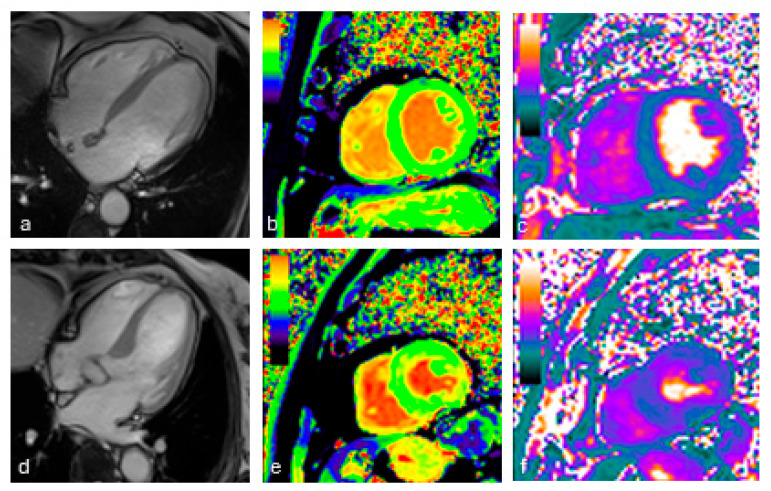
Cardiac MRI images from two COVID-19 survivors: a 62-year-old man without hypertension (**a**–**c**) and an 87-year-old woman with hypertension (**d**–**f**). For the patient without hypertension, we depict a steady-state free-precession four-chamber view (**a**), with a native T1 of 1190 ms, resulting in a normal ECV of 22.0% (**b**), and a T2 of 39 ms (**c**). For the patient with hypertension, we demonstrate a steady-state free-precession four-chamber view (**d**), with a native T1 of 1320 ms, an elevated ECV of 34.9% (**e**), and a T2 of 47 ms (**f**).

The LV end-diastolic volume index, LV ejection fraction, mass index, left atrial volume index, and native T1 did not differ between groups. The T2 did not differ between groups, but the ECV was higher in hypertensive than in non-hypertensive patients (25.0% vs. 23.6%; *p* = 0.050; Figure 2 and Figure 3; Table 5).

### 3.3. Correlations

**Figure 4:**
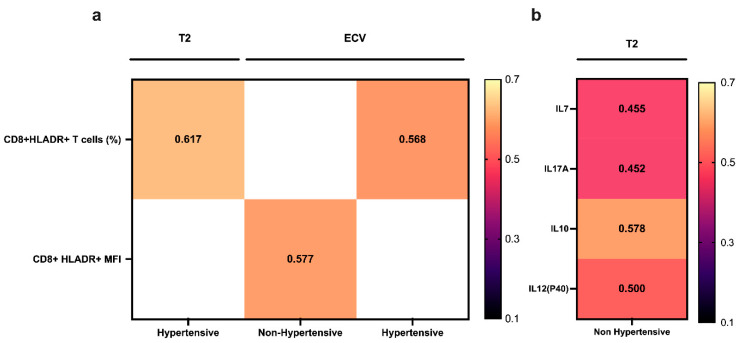
Significant correlations between immune cell subsets (**a**) and cytokines (**b**) during hospitalization for COVID-19, and T2 and ECV assessed by cardiac MRI on follow-up in patients with and without hypertension.

The total lymphocyte percentage showed a weak positive correlation (ρ = 0.386; *p* = 0.045) with the ECV in all patients. The percentage of CD8^+^ HLA-DR^+^ cells showed a moderate positive correlation with the T2 (ρ = 0.617; *p* = 0.019) and ECV (ρ = 0.568; *p* = 0.034) only in patients with hypertension. Conversely, the CD8^+^ HLA-DR^+^ MFI showed a moderate positive correlation (ρ = 0.577; *p* = 0.019) with the ECV in patients without hypertension (Table S1, Figure 4a).

Moderate positive correlations of the T2 with IL-7 (ρ = 0.455; *p* = 0.033), IL-10 (ρ = 0.578; *p* = 0.005), IL-12P40 (ρ = 0.500; *p* = 0.018), and IL-17A (ρ = 0.452; *p* = 0.035) levels were observed only in patients without hypertension. We found no correlation of any cytokine level with the ECV in hypertensive or non-hypertensive patients (Table S2, Figure 4b).

## 4. Discussion

Late cardiac involvement can have significant impacts on the physical and mental health of COVID-19 survivors. The understanding of these cardiac complications and sequelae must be improved to advance prognostic assessment and enable the development of better treatment strategies. As the dysregulation of the immune system plays a pivotal role in the mechanisms of cardiac impairment in COVID-19 [28], we assessed data on 46 immune variables acquired during hospitalization as potential markers of cardiac disease in patients with COVID-19. This study revealed cytokine and immune-cell dysregulation in hypertensive and non-hypertensive patients with COVID-19 and positive correlations of increased CD8+ T-cell activation with the T2 and ECV determined by cardiac MRI.

A very large percentage of patients in this study had increased troponin and D-dimer levels, with no difference between hypertensive and non-hypertensive groups, highlighting myocardial injury and coagulation abnormalities as frequent complications of COVID-19, regardless of the presence of hypertension. Our group previously documented the high prevalence of myocardial injury in patients hospitalized with COVID-19 (*n* = 4628), and associations of increased troponin levels with increased in-hospital mortality and adverse cardiovascular outcome rates [11]. Others have shown that increased D-dimer and fibrinogen concentrations in patients with COVID-19 are associated with poor prognosis [29].

### 4.1. Immune Biomarkers

We observed a larger percentage of CD8+ NK cells in hypertensive than in non-hypertensive patients with COVID-19 in this study, a result that aligns with our previous finding that increased CD8+ NKG2A+ MFI during hospitalization is linked to an increased risk of COVID-19 progression in hypertensive patients [7]. These findings reinforce the hypothesis that the functional exhaustion of T cells is a mechanism of immune dysregulation [30,31,32].

On the other hand, we observed reduced percentages of B cells in patients with hypertension compared to those without hypertension. B cells have been associated with cardiac and vascular diseases and can act as negative immune regulators, affecting immune-system homeostasis [33]. They are present in cardiac tissue [34] and represent about 9% of the total leukocyte population [35]. Additionally, B cells can modulate macrophage function at the cardiac level [36]. Thus, the reduced percentages of B cells observed in the present study can be associated with reduced immune modulation and increased pro-inflammatory activity as a possible underlying mechanism in hypertensive patients.

Chronic low-grade inflammation has been related to the development of cardiovascular disease and hypertension, with the involvement of the innate and adaptive immune systems [37,38]. The presence of chronic inflammation in hypertensive patients may explain our observation of increased circulating levels of IL-4, IL-5, IL-13, IL-17A, and TNF-β levels during hospitalization in patients with COVID-19 and hypertension in the present study. We did not evaluate the clinical severity of COVID-19 in this study, but in a previous study, we found that high IL-10 and IL-12 (P70) levels predicted worse clinical prognosis in hypertensive patients with COVID-19 [6].

### 4.2. Cardiac MRI Findings

Cardiovascular MRI has been used to assess cardiac involvement in COVID-19 survivors and has revealed the presence of structural and functional abnormalities [12,13,14,15,39,40,41]. Myocardial injury in the acute phase of COVID-19 (during hospitalization) may be associated with long-term cardiac involvement [39]. In the present sample, in which myocardial injury was prevalent, we also found large percentages of nonischemic LGE and pericardial enhancement. Similarly, a systematic review of data from 2954 COVID-19 survivors showed that the rate of myocardial or pericardial LGE ranged from 4% to 100%, and that myocardial LGE was predominantly nonischemic [19].

In most previous studies, T1 and T2 values have been higher in patients who had recovered from COVID-19 than in healthy controls, although no T2 elevation was detected in any patient in four of fifteen studies included in the previously mentioned systematic review [16,17,18,19,42]. In the present study, the T2 did not differ between the hypertensive and non-hypertensive groups, a comparison for which we found no previously reported data. Two other cardiac MRI studies revealed no T2 elevation during follow-up in children who presented with multisystem inflammatory syndrome during the COVID-19 pandemic [43,44]. The T2 can reflect myocardial edema, and this discrepancy in findings could be explained by measurement at different intervals after acute SARS-CoV-2 infection.

The ECV was measured in few studies included in the systematic review [19], and increased ECVs (defined by different cut-off points) were observed in COVID-19 survivors relative to controls in only two studies [14,15]. Wang et al. [45] reported increased ECVs in COVID-19 survivors (*n* = 20) relative to controls, but with no difference from values obtained during their follow-up cardiac MRI. In this study, the ECV was higher in hypertensive than in non-hypertensive patients, but we lacked previous cardiac MRI studies for these patients and thus could not examine the possibility that this elevation was linked to existing comorbidities.

### 4.3. Associations Between Biomarkers and Cardiac MRI Parameters

Late cardiac involvement of COVID-19 has been associated with immune dysregulation [46], with nonischemic patterns of myocardial delayed enhancement suggesting an inflammatory process, but the mechanism underlying this association has not been fully elucidated. Recent evidence shows that non-infectious myocardial diseases are modulated by T cells [47]. We found positive correlations between the percentage of CD8+ HLA-DR+ cells and the T2 and ECV in patients with hypertension in this study. These MRI parameters may indicate the presence of residual edema and fibrosis, respectively. In addition, CD8+ HLA-DR+ MFI showed a moderate positive correlation with the ECV in patients without hypertension. The positive correlations between ECV and activated subsets of CD8+ T cells emphasize the link between ECV and immune stimulation in patients with and without hypertension. Our group has demonstrated that these immune markers of T-cell activation are also markers of myocardial injury in hypertensive patients with COVID-19 [5].

The percentage of B cells, although reduced in patients with hypertension, was not associated with the T2 or ECV in this study. Cremonesi et al. [46] reported B-cell activation and production of antibodies against two cardiac antigens (TRIM21 and SNRNP70) in a small group of patients with delayed enhancement on 6-month follow-up MRI examination who had had high-sensitivity troponin elevation during hospitalization for COVID-19. Non-hypertensive patients in this study also showed signs of immune dysregulation, with moderate positive correlations of the T2 with the IL-7, IL-10, IL-12P40, and IL-17A levels. These findings implicate immune dysregulation as a probable cause of myocardial edema in these patients. Interestingly, we observed that the correlations among immune subsets and cytokines and remodeling biomarkers (T2 and ECV) were different in patients with and without hypertension, suggesting a different immune profile between groups.

This study has some limitations. The lack of previous cardiac MRI of the patients limits our knowledge regarding the presence of previous myocardial LGE, T2, and ECV values. The presence of coronary disease was not investigated systematically, only according to clinical criteria during hospitalization. Also, different time intervals between hospitalization, blood withdrawal, and cardiac MRI among patients may affect the results. Finally, the fact that less than 50% of the patients consented to return for MRI could be a potential bias; nevertheless, with the strategy of stratifying and comparing patients with and without hypertension a potential selection bias was minimized, as both groups were balanced, except for the presence of hypertension. Future research should focus on improving the follow-up of COVID-19 survivors with cardiac symptoms or signs of cardiac inflammation and exploring new treatment strategies.

## 5. Conclusions

The findings of this study support the hypothesis that the dysregulation of the immune system is involved in the development of cardiac complications among COVID-19 survivors, particularly in patients with hypertension who showed a different immune profile, with reduced B-cell and increased NK percentages, and higher ILs levels. The association between ECV and activated CD8+ T-cell subsets highlights a link between immune pathways and myocardial fibrosis, suggesting that immune dysregulation may intensify fibrotic processes in both hypertensive and non-hypertensive patients. The observed correlation between T2, which reflects edema, and activated CD8+ T-cell subsets exclusively in hypertensive patients could indicate a longer inflammatory response duration in this group.

## Figures and Tables

**Table 1:** Baseline characteristics of patients hospitalized for COVID-19 according to hypertensive status.

Characteristics	Total		Hypertension				χ^2^ Test *p*-Value
			No		Yes		
	*n*	(%)	*n*	(%)	n	(%)	
Sex							
Male	34	(73.9)	17	(68.0)	17	(81.0)	0.502
Female	12	(26.1)	8	(32.0)	4	(19.0)	
Age							
18–59 years	24	(52.2)	17	(68.0)	7	(33.3)	**0.037**
60–87 years	22	(47.8)	8	(32.0)	14	(66.7)	
Diabetes							
No	34	(73.9)	23	(92.0)	11	(52.4)	**0.005**
Yes	12	(26.1)	2	(8.0)	10	(47.6)	
Cardiac disease ^a^							
No	37	(80.4)	23	(92.0)	14	(66.7)	0.059
Yes	9	(19.6	2	(8.0)	7	(33.3)	
Pulmonary disease ^b^							
No	43	(93.5)	25	(100.0)	18	(85.7)	0.088
Yes	3	(6.5)	0	(0.0)	3	(14.3)	
Obesity							
No	25	(54.3	15	(60.0)	10	(47.6)	0.553
Yes	21	(45.7)	10	(40.0)	11	(52.4)	
Myocardial injury ^c^							
No	12	(26.1)	8	(32.0)	4	(19.0)	0.502
Yes	34	(73.9)	17	(68.0)	17	(81.0)	
D-dimer							
No	8	(17.4)	4	(16.0)	6	(28.6)	0.475
Yes	36	(78.3)	21	(84.0)	15	(71.4)	
TOTAL	46	(100.0)	25	(100.0)	21	(100.0)	

^a^ Chronic heart disease, heart failure, and coronary and valvular diseases. ^b^ Chronic lung disease and asthma. ^c^ Myocardial injury, defined by increased troponin levels. COVID-19, 2019 coronavirus disease. Bold values indicate *p* ≤ 0.05.

**Table 2:** Immune markers of 46 patients hospitalized for COVID-19.

Immune Markers	*n*	Mean	SD	Minimum	Median	Maximum	*p*-Value *
Monocytes (%)	30	5.9	5.5	0.1	4.4	18.3	0.236
B lymphocytes (%)	30	12.7	10.2	2.3	9.5	45.8	0.034
NK lymphocytes (%)	30	19.9	16.1	3.8	14.4	75.0	0.273
NK NKG2A (%)	30	32.2	13.7	6.5	32.2	58.2	0.987
NK NKG2A MFI	30	3116.6	1012.8	1317.2	3134.4	5293.2	0.959
Total lymphocytes (%)	30	39.1	21.5	1.2	41.8	69.5	0.629
T lymphocytes (%)	30	64.9	16.2	22.3	67.9	87.6	0.735
CD4+ T cells (%)	30	57.0	16.3	23.2	62.1	80.4	0.486
CD8+ T cells (%)	30	34.9	13.2	1.6	31.7	63.3	0.660
CD8+ CD38+ T cells (%)	30	22.7	18.8	4.0	16.6	77.3	0.202
CD8+ CD38+ (MFI)	30	2653.3	1114.8	1372.0	2240.1	5075.7	0.127
CD8+ HLA-DR+ T cells (%)	30	8.9	16.9	0.5	4.5	88.8	0.002
CD8+ HLA-DR+ MFI	30	7210.0	3222.0	2239.1	6877.2	13,499.4	0.519
CD8+ NKG2A cells (%)	30	6.4	5.2	0.0	5.7	25.6	0.643
CD8+ NKG2A MFI	30	2965.9	1086.1	0.0	2937.7	4605.0	0.934
CD8+ HLA-DR+ CD38− cells (%)	30	3.3	8.4	0.0	1.0	45.4	0.001
CD8+ HLA-DR+ CD38+ cells (%)	30	5.6	8.7	0.3	3.1	43.4	0.006
CD8+ HLA-DR− CD38+ cells (%)	30	17.1	18.6	2.0	11.4	74.1	0.056
CD8+ HLA-DR− CD38− cells (%)	30	73.9	22.0	8.9	82.2	95.7	0.056
EGF	39	28.4	46.1	0.5	8.7	195.6	0.002
Eotaxin	39	141.7	100.4	1.9	118.0	539.4	0.514
GCSF	39	315.0	999.5	0.0	51.4	4500.1	<0.001
GMCSF	39	50.2	251.7	0.1	3.6	1574.8	<0.001
IFNa2	39	95.0	208.0	0.0	55.2	1305.8	<0.001
IL1beta	39	12.2	51.4	0.1	0.5	309.4	<0.001
ILRA	39	100.4	265.6	0.1	33.4	1664.6	<0.001
IL-2	39	14.3	65.4	0.0	0.5	400.5	<0.001
IL-3	39	4.3	18.3	0.0	0.0	90.0	<0.001
IL-4	39	465.4	1.505.0	0.0	0.3	8133.5	<0.001
IL-5	39	23.5	84.0	0.1	1.5	516.2	<0.001
IL-6	39	39.5	66.7	0.0	7.3	244.2	0.001
IL-7	39	21.9	70.7	0.0	5.6	440.8	<0.001
IL-17A	39	28.6	138.7	0.0	1.5	865.6	<0.001
IL-8	39	28.9	45.3	0.1	11.8	216.8	0.002
IL-10	39	117.0	336.6	0.2	14.9	1710.5	<0.001
IL-13	39	33.4	95.2	0.0	0.9	513.6	<0.001
IL-15	39	21.2	88.9	0.2	3.3	555.3	<0.001
IL-12P70	39	58.8	326.3	0.0	2.0	2040.9	<0.001
IL-12P40	39	39.3	188.4	0.0	2.4	1177.3	<0.001
IP10	39	2629.0	2870.4	0.0	1820.0	13,282.5	0.115
MCP1	39	535.1	669.9	0.2	257.3	3023.7	0.006
MIP1beta	39	20.9	41.1	0.0	8.6	237.6	<0.001
MIP1alpha	39	14.4	33.8	0.0	3.9	160.6	<0.001
TNFalpha	39	29.9	70.2	0.1	8.1	326.1	<0.001
TNFbeta	39	47.8	118.1	0.0	3.7	579.7	<0.001
VEGF	39	102.7	120.6	0.0	50.9	536.1	0.083

* Kolmogorov–Smirnov test. Cytokine values are presented as pg/mL. COVID-19, 2019 coronavirus disease; SD, standard deviation; NK, natural killer; NKG2A, natural killer group 2-member A; MFI, mean fluorescent intensity; HLA-DR, human leukocyte antigen DR isotope; EGF, epidermal growth factor; GCSF, granulocyte colony-stimulating factor; GMCSF, granulocyte–macrophage colony-stimulating factor; IFN, interferon; IL, interleukin; IP-10, interferon gamma–inducible protein 10; MCP-1, monocyte chemoattractant protein 1; MIP, macrophage inflammatory protein; TNF, tumor necrosis factor; VEGF, vascular endothelial growth factor.

**Table 3:** Immune cell subsets in patients hospitalized for COVID-19 according to hypertensive status.

Immune Cells	Hypertension	Mean	SD	Minimum	Median	Maximum	*p*-Value *
Monocytes (%)	No	5.8	5.1	0.3	4.0	18.3	0.728
	Yes	6.0	6.1	0.1	4.8	17.8	
B lymphocytes (%)	No	14.6	9.1	2.9	11.6	36.9	0.043
	Yes	10.5	11.2	2.3	6.9	45.8	
NK lymphocytes (%)	No	12.5	7.8	3.8	10.7	31.5	0.008
	Yes	28.3	19.0	3.9	27.9	75.0	
NK NKG2A (%)	No	34.6	13.7	7.2	31.8	58.2	0.448
	Yes	29.5	13.7	6.5	33.0	58.2	
NK NKG2A MFI	No	3018.4	910.4	1545.0	3184.0	4571.9	0.637
	Yes	3228.8	1143.0	1317.2	3059.7	5293.2	
Lymphocytes (%)	No	37.0	23.0	5.4	40.4	68.5	0.637
	Yes	41.5	20.3	1.2	48.8	69.5	
T lymphocytes (%)	No	67.5	13.7	41.2	68.4	87.6	0.448
	Yes	62.0	18.7	22.3	64.7	85.6	
CD4+ T cells (%)	No	52.6	17.6	23.2	56.5	78.7	0.142
	Yes	62.1	13.5	32.4	65.6	80.4	
CD8+ T cells (%)	No	38.1	11.6	18.9	37.0	57.8	0.110
	Yes	31.1	14.3	1.6	29.4	63.3	
CD8+ CD38+ T cells (%)	No	26.0	18.6	6.3	21.0	64.8	0.224
	Yes	19.0	18.9	4.0	13.6	77.3	
CD8+ CD38+ (MFI)	No	2720.6	952.3	1881.3	2449.9	5075.7	0.120
	Yes	2576.2	1309.3	1372.0	1.852.9	4977.5	
CD8+ HLA-DR+ T cells (%)	No	10.4	21.3	0.5	4.4	88.8	0.854
	Yes	7.3	10.4	0.8	4.5	41.1	
CD8+ HLA-DR+ MFI	No	7512.3	3093.3	2780.7	6877.2	13,499.4	0.498
	Yes	6864.6	3446.3	2239.1	6361.7	12,096.3	
CD8+ NKG2A cells (%)	No	6.1	3.8	1.3	5.7	13.6	0.759
	Yes	6.6	6.6	0.0	5.1	25.6	
CD8+ NKG2A MFI	No	3031.8	873.2	1635.7	2807.8	4581.0	0.984
	Yes	2890.5	1319.2	0.0	3196.4	4605.0	
CD8+ HLA-DR+ CD38− cells (%)	No	4.0	11.1	0.0	0.8	45.4	0.637
	Yes	2.5	3.7	0.0	1.0	13.3	
CD8+ HLA-DR+ CD38+ cells (%)	No	6.3	10.2	0.4	3.9	43.4	0.552
	Yes	4.8	7.0	0.3	2.8	27.8	
CD8+ HLA-DR− CD38+ cells (%)	No	19.7	19.3	2.3	11.6	62.8	0.498
	Yes	14.2	18.1	2.0	10.1	74.1	
CD8+ HLA-DR− CD38− cells (%)	No	69.9	23.7	8.9	77.1	92.4	0.224
	Yes	78.5	19.7	22.7	85.7	95.7	

* Mann–Whitney test. COVID-19, 2019 coronavirus disease; SD, standard deviation; NK, natural killer; NKG2A, natural killer group 2-member A; MFI, mean fluorescent intensity; HLA-DR, human leukocyte antigen DR isotope.

**Table 4:** Cytokines in patients hospitalized for COVID-19 according to hypertensive status.

Cytokines	Hypertension	Mean	SD	Minimum	Median	Maximum	*p*-Value *
EGF	No	20.7	36.5	0.5	8.6	158.5	0.362
	Yes	38.3	55.8	1.8	10.4	195.6	
Eotaxin	No	120.6	77.0	1.9	98.8	321.9	0.172
	Yes	169.0	121.5	25.9	138.0	539.4	
GCSF	No	53.2	55.2	0.0	25.0	186.5	0.124
	Yes	653.8	1467.1	7.0	62.8	4500.1	
GMCSF	No	4.5	5.1	0.1	2.4	17.7	0.067
	Yes	109.4	379.3	0.8	5.1	1574.8	
IFNa2	No	60.7	67.8	0.0	27.5	222.5	0.255
	Yes	139.4	304.9	4.1	59.3	1305.8	
IL1beta	No	1.3	1.8	0.1	0.4	5.6	0.333
	Yes	26.3	76.7	0.1	1.0	309.4	
ILRA	No	58.5	69.1	0.1	34.2	276.5	0.408
	Yes	154.7	394.6	9.6	30.4	1664.6	
IL-2	No	1.0	1.4	0.0	0.4	4.8	0.124
	Yes	31.5	97.9	0.1	0.9	400.5	
IL-3	No	0.1	0.1	0.0	0.0	0.5	0.834
	Yes	9.8	27.1	0.0	0.0	90.0	
IL-4	No	130.5	383.1	0.0	0.1	1561.0	0.048
	Yes	898.8	2198.4	0.0	4.8	8133.5	
IL-5	No	7.1	18.5	0.1	1.2	80.4	0.020
	Yes	44.7	124.4	0.6	2.2	516.2	
IL-6	No	25.2	49.6	0.0	4.1	206.1	0.124
	Yes	57.9	82.0	0.0	21.1	244.2	
IL-7	No	7.6	8.9	0.0	4.8	35.8	0.163
	Yes	40.5	105.4	0.7	7.8	440.8	
IL-17A	No	1.6	2.0	0.0	1.1	7.0	0.010
	Yes	63.6	208.4	0.5	2.4	865.6	
IL-8	No	21.0	32.7	0.1	8.6	120.4	0.267
	Yes	39.2	57.2	0.6	16.6	216.8	
IL-10	No	49.2	129.5	0.2	14.3	624.0	0.279
	Yes	204.6	482.2	7.1	19.0	1710.5	
IL-13	No	19.7	63.3	0.0	0.8	268.0	0.034
	Yes	51.0	125.2	0.0	1.4	513.6	
IL-15	No	3.4	2.5	0.2	3.0	10.7	0.087
	Yes	44.1	133.4	0.7	5.3	555.3	
IL-12P70	No	1.9	1.6	0.0	1.8	5.2	0.081
	Yes	132.4	492.7	0.2	2.4	2040.9	
IL-12P40	No	3.9	5.8	0.0	2.2	24.2	0.059
	Yes	85.1	283.4	0.0	5.1	1177.3	
IP10	No	2921.5	3276.4	0.0	2151.5	13,282.5	0.685
	Yes	2250.5	2282.4	81.8	1497.0	8956.8	
MCP1	No	502.1	692.9	0.2	242.8	3023.7	0.510
	Yes	577.7	657.6	43.2	361.5	2598.3	
MIP1beta	No	12.9	18.6	0.0	7.9	92.7	0.221
	Yes	31.3	58.0	3.0	13.0	237.6	
MIP1alpha	No	10.8	33.6	0.0	3.3	160.6	0.172
	Yes	19.0	34.5	0.0	5.2	129.8	
TNFalpha	No	12.9	14.5	0.1	7.9	54.0	0.305
	Yes	51.8	102.6	1.5	9.4	326.1	
TNFbeta	No	19.6	48.7	0.0	2.0	188.5	0.013
	Yes	84.2	165.9	0.3	6.7	579.7	
VEGF	No	84.3	84.4	0.0	47.3	259.0	0.475
	Yes	126.4	155.4	11.7	69.9	536.1	

* Mann–Whitney test. Cytokine values are presented as pg/mL. COVID-19, 2019 coronavirus disease; SD, standard deviation; EGF, epidermal growth factor; GCSF, granulocyte colony-stimulating factor; GMCSF, granulocyte–macrophage colony-stimulating factor; IFN, interferon; IL, interleukin; IP-10, interferon gamma–inducible protein 10; MCP-1, monocyte chemoattractant protein 1; MIP, macrophage inflammatory protein; TNF, tumor necrosis factor; VEGF, vascular endothelial growth factor.

**Table 5:** Cardiac MRI parameters in COVID-19 survivors.

Cardiac MRI	Hypertension	Mean	SD	Minimum	Median	Maximum	*p*-Value *
LV EDVI, mL/m^2^	No	72.5	17.9	36.0	73.0	117.0	0.537
	Yes	69.5	17.6	41.0	68.0	104.0	
	Total	71.1	17.6	36.0	70.5	117.0	
LV EF, %	No	62.3	7.3	41.0	62.0	79.0	0.833
	Yes	62.5	6.1	43.0	61.0	73.0	
	Total	62.4	6.7	41.0	62.0	79.0	
LVMI, g/m^2^	No	96.2	31.4	39.0	100.0	155.0	0.107
	Yes	119.7	45.9	76.0	108.0	268.0	
	Total	107.0	40.0	39.0	104.5	268.0	
LAVI, mL/m^2^	No	28.6	4.9	20.0	28.0	37.0	0.799
	Yes	32.8	13.6	15.0	28.0	72.0	
	Total	30.5	10.0	15.0	28.0	72.0	
Native T1, ms	No	1250.2	134.5	1135.0	1233.0	1831.0	0.182
	Yes	1259.2	119.2	1116.0	1249.0	1736.0	
	Total	1254.3	126.4	1116.0	1235.5	1831.0	
T2, ms	No	41.3	2.8	36.0	41.0	50.0	0.697
	Yes	41.7	2.7	38.0	41.0	47.0	
	Total	41.5	2.7	36.0	41.0	50.0	
ECV, %	No	23.6	3.1	18.8	23.0	34.9	0.050
	Yes	25.0	3.2	20.8	25.0	34.9	
	Total	24.2	3.2	18.8	23.3	34.9	

* Mann–Whitney test. MRI, magnetic resonance imaging; COVID-19, 2019 coronavirus disease; SD, standard deviation; LV, left-ventricular; EDVI, end-diastolic volume index; EF, ejection fraction; LVMI, left-ventricular mass index; LAVI, left atrial volume index; ECV, extracellular volume. Bold values indicate *p* ≤ 0.05.

## Data Availability

The raw data supporting the conclusions of this article will be made available by the authors, without undue reservation.

## Supplementary Materials

The following supporting information can be downloaded at: https://www.mdpi.com/article/10.3390/jcm13237317/s1, Table S1: Correlation between immune cell subsets during hospitalization for COVID-19, and T2 and ECV assessed by cardiac MRI on follow up, in patients with and without hypertension. Table S2. Correlation between cytokines during hospitalization for COVID-19, and T2 and ECV assessed by cardiac MRI on follow up, in patients with and without hypertension.
