# Supplementary material for: Immune Profile and MRI-Detected Cardiac Fibrosis and Edema in Hypertensive and Non-Hypertensive Patients with COVID-19
Source: J Clin Med. 2024 Dec 2;13(23):7317. doi: 10.3390/jcm13237317 (PMC11642154; doi:10.3390/jcm13237317)
Supplement: Supplementary file 1 [file jcm-13-07317-s001.zip › jcm-3285863-supplementary.pdf]

Supplementary Material

Immune imprinting as predictor of MRI-detected cardiac fibrosis and edema in patients with hypertension and COVID-19

Short title/running head: Immune imprinting for cardiac fibrosis and edema in COVID-19

Renata Moll-Bernardes 1, Gabriel C. Camargo 1, Andréa Silvestre-Sousa 1,2, Julia Machado Barroso 1, Juliana R. Ferreira 1,3, Mariana B. Tortelly 1,3, Adriana L. Pimentel 1,3, Ana Cristina B. S. Figueiredo 1,3, Eduardo B. Schaustz 1, José Carlos P. Secco 1, Sergio C. Fortier 1, Narendra Vera 4, Luciana Conde 4, Mauro Jorge Cabral-Castro 5,6,, Denilson C. Albuquerque 1,7, Paulo H. Rosado-de-Castro 1, Martha V. T. Pinheiro 1,3, Olga F. Souza 1,3, Ronir R. Luiz 1,8 and Emiliano Medei 1,9,*

Affiliations:

1 D'Or Institute for Research and Education, Rio de Janeiro 22281-100, Brazil; renata.moll@idor.org (R.M.-B.); gabccamargo@gmail.com (G.C.C.); andreasilvestre0203@gmail.com (A.S.-S.); machadobarroso.julia@gmail.com (J.M.B.); julianacp2@gmail.com (J.R.F.); mariana.tortelly@niteroidor.com.br (M.B.T.); adriana.munford@niteroidor.com.br (A.L.P.); ana.figueiredo@rededor.com.br (A.C.B.S.F.); eduardo.schaustz@idor.org (E.B.S.); jose.secco@idor.org (J.C.P.S.); sergio.fortier@rededor.com.br (S.C.F.); denilsoncalbuquerque@gmail.com (D.C.A.); paulo.rosado@idor.org (P.H.R.-d.-C.); martha.pinheiro@rededor.com.br (M.V.T.P.); olga.souza@rededor.com.br (O.F.S.); ronir@iesc.ufrj.br (R.R.L.)

2 Evandro Chagas National Institute of Infectious Disease, Oswaldo Cruz Foundation, Rio de Janeiro 21040-360, Brazil;

3 Cardiology and Internal Medicine Department, Rede D'Or São Luiz, Rio de Janeiro 22281-100, Brazil

4 Institute of Biophysics Carlos Chagas Filho, Federal University of Rio de Janeiro (UFRJ), Rio de Janeiro 21941-902, Brazil; vera.narendra@gmail.com (N.V.); conde_luciana@hotmail.com (L.C.)

5 Institute of Microbiology Paulo de Góes, UFRJ, Rio de Janeiro 21941-902, Brazil; mauro-jorge@micro.ufrj.br

6 Department of Pathology, Faculty of Medicine, Fluminense Federal University, Niterói, Rio de Janeiro 24033-900, Brazil

7 Cardiology Department, Rio de Janeiro State University, Rio de Janeiro 20551-030, Brazil

8 Institute for Studies in Public Health—IESC, UFRJ, Rio de Janeiro 21941-598, Brazil

9 National [illegible]FRJ, Rio de Janeiro 21941-902, Brazil

* Correspondence: emedei70@biof.ufrj.br; Tel./Fax: +55-21-3938-0370

Corresponding author: Emiliano Medei. e-mail: emedei70@biof.ufrj.br

Table S1. Correlation between immune cell subsets during hospitalization for COVID-19, and T2 and ECV assessed by cardiac MRI on follow up, in patients with and without hypertension.

Immune cell subsets	Spearman correlation	T2 (ms)			ECV (%)		
		All	Non Hypertensive	Hypertensive	All	Non Hypertensive	Hypertensive
Monocytes (%)	ρ	-0.044	0.010	-0.089	-0.046	0.007	0.062
	p-value	0.819	0.969	0.763	0.811	0.978	0.834
B lymphocytes (%)	ρ	-0.242	-0.496	0.075	-0.231	-0.031	-0.343
	p-value	0.198	0.051	0.798	0.219	0.910	0.230
NK lymphocytes (%)	ρ	0.023	-0.362	0.269	0.181	-0.015	0.196
	p-value	0.904	0.169	0.353	0.339	0.957	0.502
NK NKG2A (%)	ρ	-0.347	-0.353	-0.504	-0.006	0.182	-0.277
	p-value	0.060	0.180	0.066	0.973	0.499	0.337
NK NKG2A MFI	ρ	0.041	0.000	-0.091	-0.012	-0.087	0.110
	p-value	0.829	1.000	0.757	0.949	0.749	0.708
Lymphocytes (%)	ρ	0.057	-0.132	0.253	**0.368**	0.344	0.238
	p-value	0.766	0.625	0.383	**0.045**	0.192	0.413
T lymphocytes (%)	ρ	-0.015	0.266	-0.295	0.071	0.185	0.018
	p-value	0.935	0.319	0.305	0.711	0.492	0.952
CD4+ T cells (%)	ρ	-0.005	-0.228	0.162	0.000	-0.038	-0.075
	p-value	0.978	0.396	0.580	0.999	0.888	0.799
CD8+ T cells (%)	ρ	-0.014	0.214	-0.133	-0.010	0.065	0.059
	p-value	0.941	0.425	0.650	0.956	0.812	0.840
CD8+ CD38+ T cells (%)	ρ	0.081	0.320	-0.011	0.042	0.016	0.097
	p-value	0.669	0.227	0.970	0.826	0.953	0.742
CD8+ CD38+ (MFI)	ρ	-0.058	-0.021	-0.213	-0.003	0.341	-0.009
	p-value	0.762	0.939	0.464	0.986	0.196	0.976
CD8+ HLADR+ T cells (%)	ρ	0.249	-0.101	**0.617**	0.068	-0.316	**0.568**
	p-value	0.185	0.709	**0.019**	0.720	0.232	**0.034**
CD8+ HLADR+ MFI	ρ	-0.248	-0.003	-0.488	-0.055	**0.577**	-0.400

	p-valor	0.187	0.991	0.076	0.772	**0.019**	0.156
CD8+ NKG2A cells (%)	ρ	-0.209	-0.060	-0.324	-0.056	0.222	-0.224
	p-valor	0.269	0.827	0.258	0.768	0.408	0.441
CD8+ NKG2A MFI	ρ	0.125	0.320	-0.211	0.248	0.155	0.183
	p-valor	0.509	0.227	0.469	0.186	0.568	0.532
CD8+ HLADR+ CD38- cells (%)	ρ	0.181	-0.113	0.479	0.000	-0.255	0.385
	p-valor	0.338	0.676	0.083	0.999	0.341	0.174
CD8+ HLADR+ CD38+ cells(%)	ρ	0.204	-0.058	0.506	0.071	-0.191	0.493
	p-valor	0.279	0.831	0.065	0.708	0.478	0.073
CD8+ HLADR- CD38+ cells (%)	ρ	-0.004	0.229	-0.184	0.051	0.266	-0.246
	p-valor	0.981	0.393	0.528	0.791	0.319	0.396
CD8+ HLADR- CD38- cells (%)	ρ	-0.128	-0.260	-0.058	-0.075	0.031	-0.163
	p-valor	0.500	0.330	0.845	0.694	0.910	0.578

COVID-19, 2019 coronavirus disease; ECV, extracellular volume; MRI, magnetic resonance imaging; NK, natural killer; NKG2A, natural killer group 2-member A ; MFI, mean fluorescent intensity; HLA-DR, human leukocyte antigen DR isotope.

Bold values indicate significant correlation.

Table S2. Correlation between cytokines during hospitalization for COVID-19, and T2 and ECV assessed by cardiac MRI on follow up, in patients with and without hypertension.

Cytokines	**Spearman correlation**	**T2 ms**			**ECV**		
		**All**	**Non Hypertensive**	**Hypertensive**	**All**	**Non Hypertensive**	**Hypertensive**
EGF	ρ	0.014	0.132	-0.091	0.011	-0.084	0.010
	p-value	0.933	0.560	0.729	0.945	0.709	0.970
Eotaxin	ρ	-0.044	0.050	-0.113	-0.175	-0.308	-0.076
	p-value	0.790	0.824	0.666	0.286	0.163	0.771
GCSF	ρ	0.135	0.326	-0.072	0.053	-0.205	0.192
	p-value	0.412	0.139	0.783	0.749	0.359	0.461
GMCSF	ρ	0.206	0.297	-0.017	0.083	-0.051	0.047
	p-value	0.208	0.179	0.947	0.615	0.822	0.859
IFNa2	ρ	0.218	0.346	0.048	0.139	-0.093	0.472
	p-value	0.182	0.115	0.853	0.399	0.680	0.056
IL1beta	ρ	0.101	0.413	-0.190	0.054	0.029	0.007
	p-value	0.539	0.056	0.465	0.745	0.900	0.978
ILRA	ρ	0.160	0.307	-0.007	0.054	-0.265	0.366
	p-value	0.337	0.164	0.978	0.746	0.234	0.163
IL2	ρ	0.109	0.325	-0.174	0.064	-0.064	0.108
	p-value	0.509	0.140	0.504	0.699	0.778	0.680
IL3	ρ	0.001	-0.104	0.045	0.005	-0.130	0.267
	p-value	0.995	0.644	0.864	0.977	0.565	0.300
IL4	ρ	0.149	0.175	0.016	0.258	0.160	0.174
	p-value	0.366	0.435	0.953	0.114	0.477	0.505
IL5	ρ	0.073	0.105	-0.035	0.096	0.047	0.027
	p-value	0.659	0.643	0.894	0.560	0.835	0.918
IL6	ρ	0.029	0.247	-0.224	0.096	-0.029	0.027
	p-value	0.859	0.267	0.388	0.560	0.898	0.918

IL7	ρ	0.184	**0.455**	-0.048	0.098	-0.059	0.170
	p-value	0.263	**0.033**	0.853	0.552	0.796	0.515
IL17A	ρ	0.179	**0.452**	-0.246	0.016	-0.118	-0.003
	p-value	0.282	**0.035**	0.358	0.922	0.602	0.991
IL8	ρ	-0.052	0.237	-0.344	-0.094	-0.270	-0.020
	p-value	0.754	0.289	0.176	0.567	0.224	0.940
IL10	ρ	0.232	**0.578**	-0.088	0.287	0.225	0.270
	p-value	0.155	**0.005**	0.736	0.077	0.315	0.294
IL13	ρ	0.097	0.170	-0.087	0.135	0.021	0.049
	p-value	0.559	0.449	0.740	0.413	0.925	0.851
IL15	ρ	-0.066	0.225	-0.330	-0.035	-0.175	-0.014
	p-value	0.691	0.313	0.196	0.832	0.437	0.959
IL12P70	ρ	0.238	0.344	0.077	0.131	-0.121	0.268
	p-value	0.145	0.117	0.769	0.427	0.592	0.299
IL12P40	ρ	0.248	**0.500**	-0.068	0.168	-0.042	0.194
	p-value	0.128	**0.018**	0.794	0.306	0.853	0.455
IP10	ρ	0.098	0.145	0.077	0.133	0.054	0.204
	p-value	0.551	0.519	0.769	0.419	0.812	0.432
MCP1	ρ	0.101	0.117	0.044	-0.059	-0.449	0.197
	p-value	0.541	0.605	0.868	0.723	0.036	0.450
MIP1BETA	ρ	0.081	0.121	0.037	0.272	0.214	0.248
	p-value	0.625	0.591	0.887	0.094	0.338	0.337
MIP1ALFA	ρ	0.071	0.415	-0.368	-0.017	-0.089	-0.059
	p-value	0.666	0.055	0.146	0.917	0.694	0.822
TNFAlfa	ρ	0.109	0.202	0.002	0.198	-0.081	0.396
	p-value	0.508	0.366	0.992	0.228	0.720	0.116
TNFBeta	ρ	0.159	0.385	-0.206	0.131	0.047	0.022
	p-value	0.333	0.077	0.427	0.427	0.834	0.933
VEGF	ρ	0.160	0.362	-0.073	0.035	-0.008	0.052
	p-value	0.329	0.098	0.780	0.831	0.970	0.844

COVID-19, 2019 coronavirus disease; ECV, extracellular volume; MRI, magnetic resonance imaging; EGF, epidermal growth factor; GCSF, granulocyte colony-stimulating factor; GMCSF, granulocyte-macrophage colony-stimulating factor; IFN, interferon; IL, interleukin; IP-10, interferon gamma–inducible protein 10; MCP-1, monocyte chemoattractant protein 1; MIP, macrophage inflammatory protein; TNF, tumor necrosis factor; VEGF, vascular endothelial growth factor.
Bold values indicate significant correlation.
